# *Selaginella bryopteris* Aqueous Extract Improves Stability and Function of Cryopreserved Human Mesenchymal Stem Cells

**DOI:** 10.1155/2017/8530656

**Published:** 2017-07-24

**Authors:** Abhishek Kumar Singh, Anubhuti Jha, Arindam Bit, Andrey P. Kiassov, Albert A. Rizvanov, Archit Ojha, Pooja Bhoi, Pradeep Kumar Patra, Awanish Kumar, Akalabya Bissoyi

**Affiliations:** ^1^Department of Biochemistry, University of Allahabad, Allahabad 211002, India; ^2^Department of Biotechnology, National Institute of Technology, Raipur 492010, India; ^3^Department of Biomedical Engineering, National Institute of Technology, Raipur 492010, India; ^4^Kazan Federal University, Kazan, Russia; ^5^Shona Lab, Centre for Brain Development and Repair, InStem National Centre for Biological Sciences, Bangalore, Karnataka 560065, India; ^6^Department of Biochemistry, Pt. JNM Medical College, Raipur 492001, India

## Abstract

The effective long-term cryopreservation of human mesenchymal stem cells (MSCs) is an essential prerequisite step and represents a critical approach for their sustained supply in basic research, regenerative medicine, and tissue engineering applications. Therefore, attempts have been made in the present investigation to formulate a freezing solution consisting of a combination of *Selaginella bryopteris* water-soluble extract with and without dimethyl sulfoxide (Me_2_SO) for the efficient long-term storage of human umbilical cord blood- (hUCB-) derived MSCs. The cryopreservation experiment using the formulated freezing solution was further performed with hUCB MSCs in a controlled rate freezer. A significant increase in postthaw cell viability and cell attachment of MSCs was achieved with freezing medium containing *Selaginella bryopteris* water extract along with 10% Me_2_SO as compared to the freezing medium containing Me_2_SO (10% *v*/*v*) alone. Furthermore, the decreasing apoptotic events and reactive oxygen species production along with increasing expression of heat shock proteins also confirmed the beneficial effect of *Selaginella bryopteris* water extract. The beneficial effect of *Selaginella bryopteris* water extract was validated by its ability to render postpreservation high cell viability. In conclusion, the formulated freezing solution has been demonstrated to be effective for the standardization of cryopreservation protocol for hMSCs.

## 1. Introduction

In the last few decades, the advent of stem cell research has been a promising milestone in the field of cryobiology. Mesenchymal stem cells (MSCs) are specialized cells with a wide range of multiple therapeutic properties like anti-inflammatory, antiapoptotic, antifibrotic, and immunomodulatory along with tumor tropism and differentiation [1, 2]. These cells possess an extensive array of functional properties, and an account of which has been very well documented [3, 4]. Considering various applications including versatile therapeutic nature of MSCs, their long-term storage is of grave importance. However, different groups of researchers have estimated the effect of cryopreservation on the phenotype of MSCs, but a complete effectiveness of cryopreservation protocol remains elusive [5]. Therefore, an identification of novel cryoprotectants and suitable conditions for cryopreservation is a prerequisite step and represents a critical approach.

The biopreservation of MSCs has always been a critical step in stem cell biology, and it is well established that the vitrification and controlled-rate freezing are the two prominent methods for MSC banking [6]. The novel method for MSC cryopreservation has been an ever growing platform for research and recently gained considerable importance in the scientific community. Over the last decades, a vast repertoire of cryoprotectants has been discovered that could be classified into two main categories, namely, diffusible (intracellular agents) and nondiffusible (extracellular agents). The most commonly used cryoprotectants are dimethyl sulfoxide (Me_2_SO), glycerol, 1,2-propanediol, propylene glycol, polyvinylpyrolidone, and hydroxyethyl starch [7]. However, the major issues of the gold standard cryoprotectant Me_2_SO involve its potent cellular toxicity and neurodifferentiation effects during stem cell cryopreservation [8, 9]. Deployment of intracellular agents has also notable cytotoxic side effects, for instance, lethality associated with the cooling and thawing processes [10]. Over the time, the striking challenges faced with the use of currently available cryoprotectants have led to the search of alternate measures [11–14].

*Selaginella bryopteris* is a perennial xerophytic herbaceous plant that has been pharmacologically significant for years [15], and its several bioactive compounds have been shown to have variegated therapeutic properties [16]. Moreover, it has been reported to possess antioxidant activity and growth-promoting activity in various cell types [17]. In tribal India, this herb (commonly known as Sanjeevani) is profoundly popular as a chief component of natural therapy against various disorders [18]. The contents accountable for such biological activities are flavonoids whose virtues have established Sanjeevani as a panacea in medical sciences [19]. There have been few accounts of strong activities unveiled by aqueous extracts of *Selaginella bryopteris* in variegated fields like effecting the growth of BMC2 and Sf9 cells [16]. Due to its diverse ethnopharmacological activities, we hypothesize that its resurrection properties can be subjugated towards an active cryoprotective nature.

Additionally, enormous interest has been paid to understand its ability to cryopreserve stem cells. As an alternative strategy to the current scenario of ineffective cryoprotectants, we propose to utilize the resurrection property of *Selaginella bryopteris* for the effective cryopreservation of MSCs.

## 2. Materials and Materials

### 2.1. Reagents and Consumables

All the chemicals and reagents used in this study were purchased from Sigma-Aldrich Chemical (St. Louis, MO, USA) unless otherwise stated. Alpha-minimum essential medium (*α*-MEM), fetal bovine serum (FBS), dimethyl sulfoxide (Me_2_SO), antibiotic-antimycotic solution, phalloidin Alexa Fluor 488, 2′,7′-dichlorofluorescin diacetate (DCFH-DA), and 1,4-diazabicyclo [2.2.2] octane (DABCO) were purchased from Invitrogen, USA. A LIVE/DEAD® Viability/Cytotoxicity Assay Kit was purchased from Thermo Fisher Scientific (USA). Triton X-100, ethanol, paraformaldehyde, 0.25% trypsin, and plastic wares were purchased from Himedia, India. All the primary antibodies were purchased from Abcam (CA, USA) and fluorescently tagged antibodies were purchased from BD Pharmingen (Becton Dickenson, San Jose, CA).

### 2.2. Collection of Human Umbilical Cord Blood (hUCB)

hUCB was collected from Jawaharlal Nehru Medical College, Raipur, India, with prior informed consent of parents and approval of the Institutional Ethical Committee. A blood bag containing CPD anticoagulant was used for aseptic transfer of hUCB samples to the culture laboratory.

### 2.3. Isolation and Culture of hUCB-Derived Mesenchymal Stem Cells

The mononuclear cells (MNCs) were isolated from hUCB by Ficoll-Paque density gradient centrifugation (700 ×g for 20 min). Then, the MNCs were resuspended in *α*-MEM containing 10% FBS, 2 mM glutamine, and 1% antibiotic-antimycotic solution and plated at a density of 1 × 10^6^ cells/cm^3^. Nonadherent cells were discarded after incubating culture plates at 37°C in a humidified CO_2_ (5%) incubator for 24 h. The adherent mesenchymal stem cells (MSCs) were washed thoroughly with Dulbecco's phosphate buffer saline (DPBS) and then supplemented with freshly prepared expansion medium. The culture medium was changed twice in a week, and the cells were subcultured after reaching more than 80% of confluency.

### 2.4. Characterization of MSCs

The specific surface antigens present on MSCs were characterized by a flow cytometer following the protocol earlier described by us [18, 19]. The trypsinized postthawed cells (5 × 10^5^ cells) were stained with fluorescently tagged human monoclonal antibodies against CD34, CD45, CD73, CD90, and CD105. Then, the CD markers were analyzed using a flow cytometer (Becton Dickinson and Co., San Jose, CA, USA).

### 2.5. Preparation of *Selaginella bryopteris* Leaf Extract

Leaves of *Selaginella bryopteris* were cleaned, dried in an oven at 40°C, and soaked overnight in minimal volume of water. Then, the leaves were again dried, washed twice, and further pulverized. Fine powder of the leaves (30 g) was added in 100 mL of autoclaved distilled water, and the mixture was continuously shaken for 4 h at 35°C. Further, the mixture was vortexed for 2 h and subjected to centrifugation at 3000 rpm for 1 h. Finally, the supernatant was filtered using a syringe filter (0.2 *μ*m) and stored at −20°C till further experimentation.

### 2.6. Formulation and Pretreatment of Cryoprotectant Solutions to MSCs

The culture medium of MSCs was replaced with different freezing medium formulations containing either *Selaginella bryopteris* aqueous extract (5% *v*/*v*) or Me_2_SO (10% *v*/*v*) and combination of both as a cryoprotectant in *α*-MEM medium supplemented with 15% FBS. The cryopreservation study was performed in a controlled-rate freezer following the cooling protocol as described previously [20]. After achieving −180°C temperature, the cell suspension was taken out of the freezer and stored in a liquid nitrogen tank (−196°C) for further use [21]. After 7 days of cryopreservation in liquid nitrogen, MSCs were thawed and again cultured to evaluate the effect of *Selaginella bryopteris* aqueous extract as its potential application in cryoprotection. For thawing of the cryopreserved MSCs, the cell suspension was placed in water bath (Lauda RM6) at 37–40°C followed by gradual dilution of cryoprotectant solution using prewarmed growth medium. Each batch of experiments was repeated at least three times.

### 2.7. Postthaw Culture of MSCs

The postthaw viability of cryopreserved MSCs was assayed by growing in *α*-MEM supplemented with 10% FBS, 1% antibiotic-antimycotic solution, and epidermal growth factor (10 ng/mL). The cultured MSCs were maintained in a humidified atmosphere at 37°C and 5% CO_2_ [22]. For a morphological study, the postthawed MSCs were cultured in a 6-well culture plate and observed under an inverted phase-contrast microscope (Leica Microsystem, Germany).

### 2.8. Live and Dead Cell Analysis of Postthawed MSCs

The efficiency of the freezing media was evaluated after 7 days of storage of MSCs in the liquid nitrogen. The cryopreserved MSCs were thawed and cell viability was assessed immediately after thawing. Cell viability was determined by using a LIVE/DEAD Viability/Cytotoxicity Assay Kit. The dye (5 *μ*L) was added to each sample at room temperature, and after 10 min, cells were analyzed with a flow cytometer (FACS ARIA III) equipped with FACS Diva software [23].

### 2.9. Analysis of MSC Adherence and Filamentous (F) Actin Distribution in Postthawed MSCs

The cell adherence on the surface of a coverslip was evaluated by measuring the distribution of cytoskeleton F actin in cryopreserved MSCs using phalloidin Alexa Fluor 488 staining. Briefly, postthawed cells were washed with PBS (pH 7.4), grown over a coverslip, and fixed with 4% formaldehyde solution. Then, freshly prepared phalloidin Alexa Fluor 488 solution was added onto the coverslip to stain the F actin in MSCs. Finally, the cells were washed with mounting medium (DABCO, pH 8.7) and analyzed with confocal microscopy [24].

### 2.10. Determination of Reactive Oxygen Species (ROS) Production in Postthawed MSCs

To evaluate the oxidative stress, ROS production in cryopreserved MSCs was assessed by 2′,7′-dichlorofluorescin diacetate (DCFH-DA) dye. Briefly, postthawed MSCs were allowed to be stained with DCFH-DA (20 *μ*M) dye for 30 min at 37°C in the dark. After three times washing with PBS, cells were analyzed for ROS generation using a flow cytometer (FACS Calibur, BD Biosciences, USA). At least 10,000 gated events were analyzed per sample by a flow cytometer.

### 2.11. Proliferation and Viability Analysis of Postthawed MSCs by MTT Assay

To determine the postthaw cell viability of MSCs, the MTT (3-(4,5-dimethythiazol-2-yl)-2,5-diphenyltetrazolium bromide) assay was carried out following the protocol described earlier by us [25]. In brief, the postthawed MSCs were cultured in a 96-well culture plate for 24 h. Then, 10 *μ*L of MTT solution (5 mg/mL) was added to each well followed by another 4 h of incubation at 37°C [26]. After the completion of the incubation period, acidic isopropanol was added to each well to dissolve the dark blue-colored formazan crystals. Further, absorbance of the dissolved content was taken at 570 nm using a multiwell microplate reader (Bio-Tek ELx800). The noncryopreserved MSCs were used as the control. The cell viability was expressed as a percentage of control.

### 2.12. Analysis of Apoptosis in Postthawed MSCs

An apoptosis study was carried out as a part of an evaluation of the quality of cryoprotectant solutions used for preserving MSCs [27]. The evaluation of apoptotic cell death in postthawed MSCs was done by flow cytometry (FACS Calibur, BD Biosciences, USA) using an Annexin V-FITC (fluorescein isothiocyanate) Apoptosis Detection Kit following the protocol described earlier by us [28]. After 24 h of thawing, MSCs were resuspended in 500 *μ*L PBS and allowed to be stained with 5 *μ*L Annexin V-FITC and 5 *μ*L propidium iodide for 1 h in the dark. Further, the apoptotic event in cryopreserved MSCs was quantified using a flow cytometer (FACS Calibur, BD Biosciences, USA). The noncryopreserved MSCs were used as controls for the apoptosis study.

### 2.13. Western Blot Analysis of Heat Shock Proteins (Hsp70 and Hsp90) in Postthawed MSCs

After cryopreservation in different cryoprotectant formulations, the postthawed MSCs were lysed with CelLytic M cell lysis reagent in the presence of 1X protease inhibitor cocktail. Then, the changes in expression of heat shock proteins Hsp70 and Hsp90 were studied by Western immunoblotting as described earlier [29]. In brief, an equal amount of protein (40 *μ*g) from each experimental group was electrophoresed and transferred to the PVDF membrane. The membranes were blocked with 5% nonskimmed milk and incubated with primary antibodies specific for Hsp70 (1 : 1000), Hsp90 (1 : 1000), and *β*-actin (1 : 10000). Then, the membranes were washed and incubated with secondary antibodies conjugated with horseradish peroxidase. The blots were developed and quantified using Super Signal West Fempto Chemiluminescent Substrate™ (Thermo Fisher Scientific, USA) and Bio-Rad Versa Doc™ Imaging System 4000 (Bio-Rad, PA, USA).

### 2.14. Statistical Methods

The values are given as mean ± standard deviation of three independent experiments. The statistical differences among the experimental groups were assessed by one-way analysis of variance (ANOVA) followed by post hoc Tukey's multiple comparison test using GraphPad Prism 5 (GraphPad). *p* < 0.05 was considered significant.

## 3. Results

### 3.1. Phenotypic Characterization of Purified Mesenchymal Stem Cells (MSCs)

**Figure 1:**
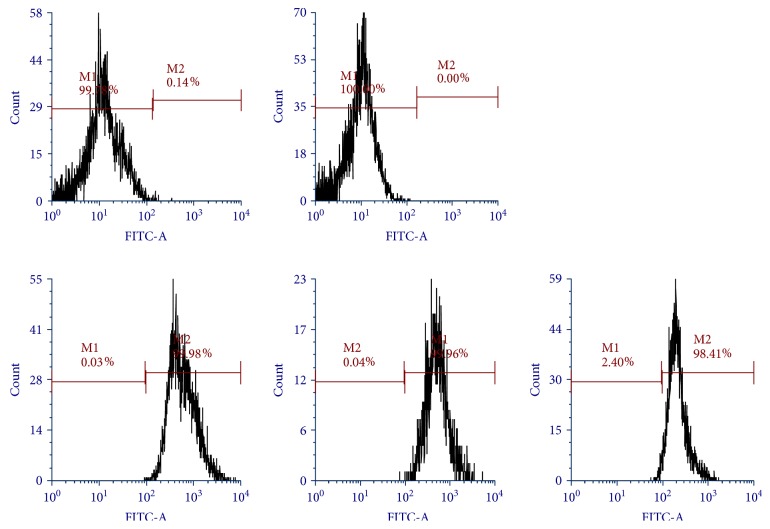
Flow cytometric characterization of MSCs stained with FITC-labelled (CD34), PE-A-labelled (CD45), APC-A-labelled (CD90), FITC-A-labelled (CD73), and PE-A-labelled (CD105) antibodies. The *x*-axis and *y*-axis indicate fluorescence intensity and cell counts, respectively.

The characterization of purified population of MSCs was carried out by measuring positive expression of MSC-specific cell surface markers, namely, CD73, CD90, and CD105, and negative expression of CD34 and CD45. The hUCB-derived mononuclear cells were initially cultured for isolation of MSCs utilizing their plastic adherence property. The adhered cells showed prominent colonies within 5 to 7 days of culture. The flow cytometry data demonstrated the positive expression of CD73 (99.98%), CD90 (98.41%), and CD105 (99.96%) and negative expression of CD34 (0.0%) and CD45 (0.14%) markers (Figure 1). The findings corroborated the high purity of MSCs in our experimental conditions.

### 3.2. Effect of *Selaginella bryopteris* Aqueous Extract on Cell Viability of Postthawed MSCs

**Figure 2:**
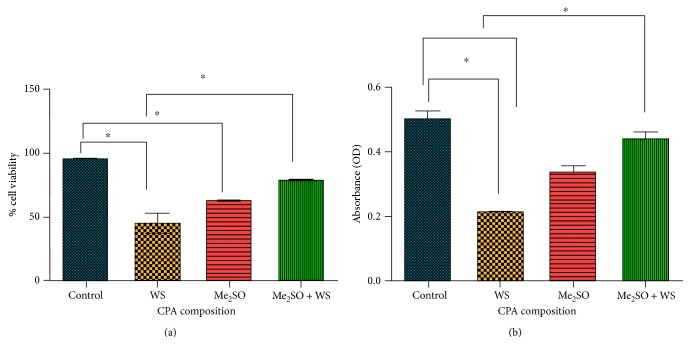
Cell viability analysis of postthawed MSCs cryopreserved with Me_2_SO, *Selaginella bryopteris* water extract, and their combination by (a) MTT assay and (b) LIVE/DEAD Viability/Cytotoxicity Assay Kit. Noncryopreserved MSCs were taken as the control. Values are given as mean ± SD of three independent experiments. ∗ represents a significance (*p* < 0.05) value.

To assess the effect of *Selaginella bryopteris* aqueous extract, Me_2_SO, and their combination on postthaw cell viability of MSCs, the MTT assay was performed and the results are highlighted in Figure 2. The data demonstrated the significant difference in cell viability of MSCs cryopreserved with *Selaginella bryopteris* aqueous extract, Me_2_SO, and their combination. The cell viability of postthawed MSCs cryopreserved with Me_2_SO (10% *v*/*v*) was found to be 60 ± 2% when compared with that of noncryopreserved control MSCs. Moreover, MSCs cryopreserved with *Selaginella bryopteris* aqueous extract (5% *v*/*v*) have cell viability of 46 ± 5% when compared with noncryopreserved control MSCs. However, the combinatorial use of *Selaginella bryopteris* aqueous extract and Me_2_SO showed significantly increased cell viability of 75 ± 3% in postthawed MSCs (Figure 2(a)). Thus, the data demonstrated that the aqueous extract of *Selaginella bryopteris* acts as a potential cryoprotectant for cryopreservation of human MSCs. Furthermore, the results also indicated the significant death of MSCs during the cryopreservation process irrespective of different cryoprotecting solutions that might be due to the physical stress caused during the freezing process. The absorbance recorded for the water extract of *Selaginella bryopteris* indicates its effectiveness slightly better than that of Me_2_SO (Figure 2(b)).

### 3.3. Effect of S*elaginella bryopteris* Aqueous Extract on Apoptotic Cell Death in Postthawed MSCs

**Figure 3:**
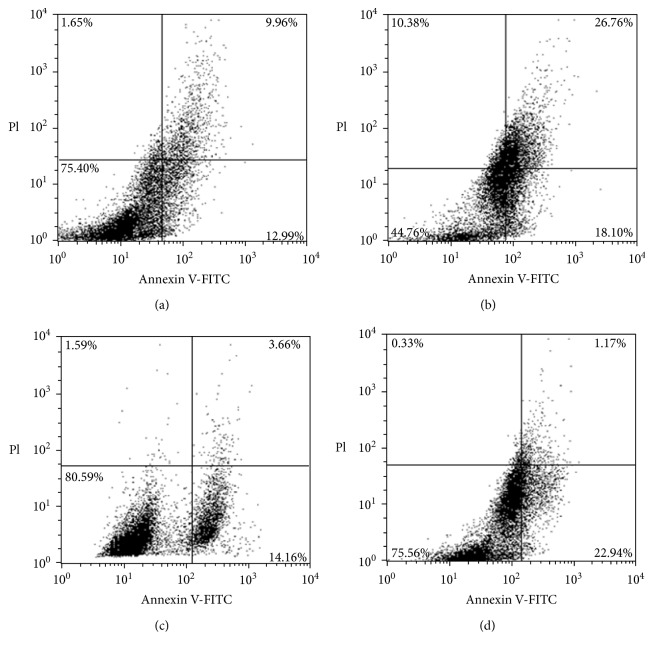
Apoptotic cell death analysis in postthawed MSCs cryopreserved with Me_2_SO, *Selaginella bryopteris* water extract, and their combination: (a) noncryopreserved control MSCs; (b) postthawed MSCs cryopreserved with the water extract of *Selaginella bryopteris* (WS); (c) postthawed MSCs cryopreserved with Me_2_SO; (d) postthawed MSCs cryopreserved with WS + Me_2_SO.

Next, we investigated the apoptotic cell death in postthawed MSCs cryopreserved with *Selaginella bryopteris* aqueous extract, Me_2_SO, and their combination using an Annexin V-FITC Apoptosis Detection Kit (Figure 3). A significant percentage of the early phase of apoptotic cell death was observed when MSCs were cryopreserved in Me_2_SO (10% *v*/*v*) and *Selaginella bryopteris* aqueous extract (5% *v*/*v*). However, the events of apoptotic cell death in postthawed MSCs were comparatively lower during cryopreservation with *Selaginella bryopteris* (18.1%) aqueous extract when compared to Me_2_SO (22.94%) as shown in Table 1. Interestingly, the use of *Selaginella bryopteris* aqueous extract and Me_2_SO in combination as freezing medium demonstrated the significant reduced percentage of early-phase apoptosis (12.99%) among all the experimental groups. Furthermore, this reduction in apoptosis was also found comparable with that of noncryopreserved control MSCs (14.16%).

### 3.4. Effect of S*elaginella bryopteris* Aqueous Extract on ROS Production in Postthawed MSCs

**Figure 4:**
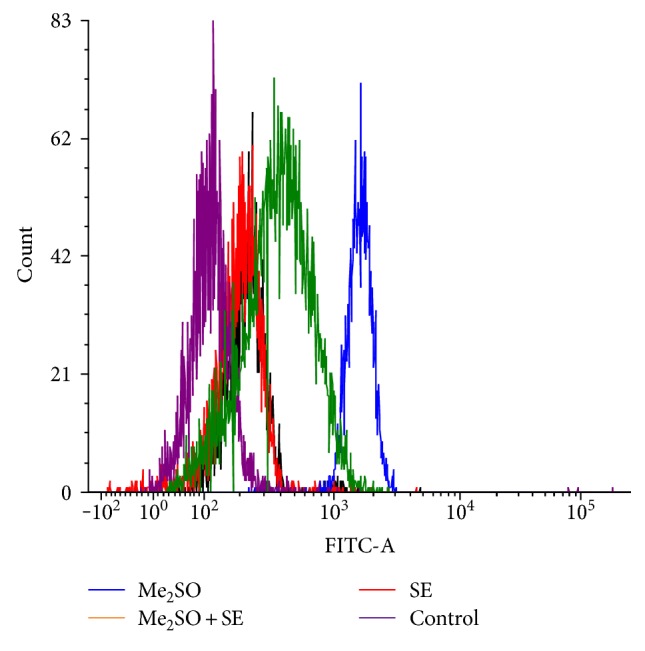
Measurement of ROS production in postthawed MSCs cryopreserved with Me_2_SO, *Selaginella bryopteris* water extract (WS), and their combination by a flow cytometer using DCFH-DA dye.

Cryopreservation causes detrimental effects on cells in terms of damage to mitochondria; hence, its freezing effect causing generation of reactive oxygen species (ROS) was analyzed. It has been widely suggested that the overproduction of ROS is responsible for cellular death. In order to understand the precise mechanism of cell death and apoptosis, we hypothesized that the overproduction of ROS may be a major contributor of cell death. Interestingly, a dramatic increase in ROS was observed in postthawed MSCs cryopreserved with *Selaginella bryopteris* water extract. However, the maximum elevation in level of ROS was observed in postthawed MSCs cryopreserved with Me_2_SO when compared to noncryopreserved control cells. The combination of *Selaginella bryopteris* water extract and Me_2_SO significantly reduced the level of ROS production that was brought closer to the level of noncryopreserved control MSCs (Figure 4).

### 3.5. Effect of S*elaginella bryopteris* Aqueous Extract on Distribution of Filamentous Actin and Cell Adherence in Postthawed MSCs

**Figure 5:**
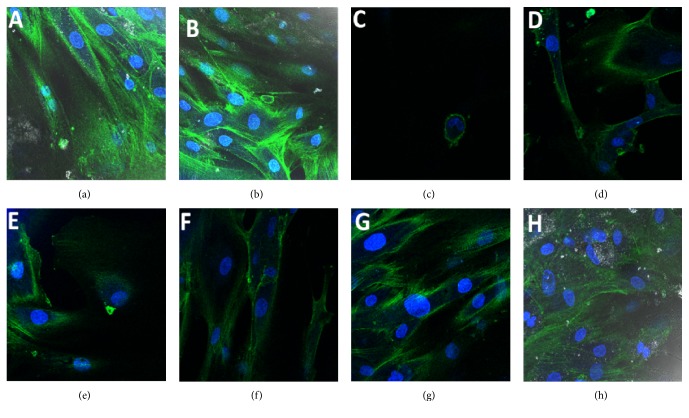
Analysis of cytoskeleton distribution in postthawed MSCs by confocal microscopy: (a) cytoskeleton distribution in noncryopreserved MSCs after 48 h of culture; (b) cytoskeleton distribution in noncryopreserved MSCs after 72 h of culture; (c) cytoskeleton distribution in postthawed MSCs cryopreserved with *Selaginella bryopteris* water extract (WS) after 48 h of culture; (d) cytoskeleton distribution in postthawed MSCs cryopreserved with *Selaginella bryopteris* water extract (WS) after 72 h of culture; (e) cytoskeleton distribution in postthawed MSCs cryopreserved with Me_2_SO after 48 h of culture; (f) cytoskeleton distribution in postthawed MSCs cryopreserved with Me_2_SO after 72 h of culture; (g) cytoskeleton distribution in postthawed MSCs cryopreserved with WS + Me_2_SO after 48 h of culture; (h) cytoskeleton distribution in postthawed MSCs cryopreserved with WS + Me_2_SO after 72 h of culture.

Fluorescence image analysis was carried out to evaluate the changes in the cytoskeleton structure of MSCs cryopreserved with different cryoprotectant solutions, namely, *Selaginella bryopteris* water extract (5% *v*/*v*), Me_2_SO (10% *v*/*v*), and their combination. The noncryopreserved MSCs showed normal fibroblast-like cell morphology and intact organization of F actin after 48 h and 72 h of culture (Figures 5(a) and 5(b)). However, *Selaginella bryopteris* water extract induced the loss of F actin distribution and impaired cell adherence in postthawed MSCs after 48 h of culture (Figure 5(c)) that was slightly regained during the progress of culture for 72 h (Figure 5(d)). The postthawed MSCs cryopreserved in Me_2_SO were also not able to recuperate their original shape even after 48 h of culture (Figure 5(e)). In addition, the freezing solution containing both *Selaginella bryopteris* water extract and Me_2_SO retained the original shape without undergoing any deformation after 48 h of culture of postthawed MSCs (Figure 5(g)). During the progress of time in culture condition after 72 h, postthawed MSCs cryopreserved in freezing medium containing *Selaginella bryopteris* water extract and Me_2_SO regained their original shape with normal distribution of F actin and cell adherence (Figure 5(h)). MSCs frozen in Me_2_SO and *Selaginella bryopteris* water extract became long shuttle-shaped. Moreover, MSCs that were cryopreserved with *Selaginella bryopteris* aqueous extract, Me_2_SO, and their combination had almost similar cell morphologies. Additionally, MSCs cryopreserved with *Selaginella bryopteris* showed more spread out and cell-cell contacts as compared to the cells cryopreserved with Me_2_SO. However, the cells still had more rounded shape when compared to noncryopreserved cells.

### 3.6. Effect of S*elaginella bryopteris* Aqueous Extract on the Expression of Heat Shock Proteins in Postthawed MSCs

**Figure 6:**
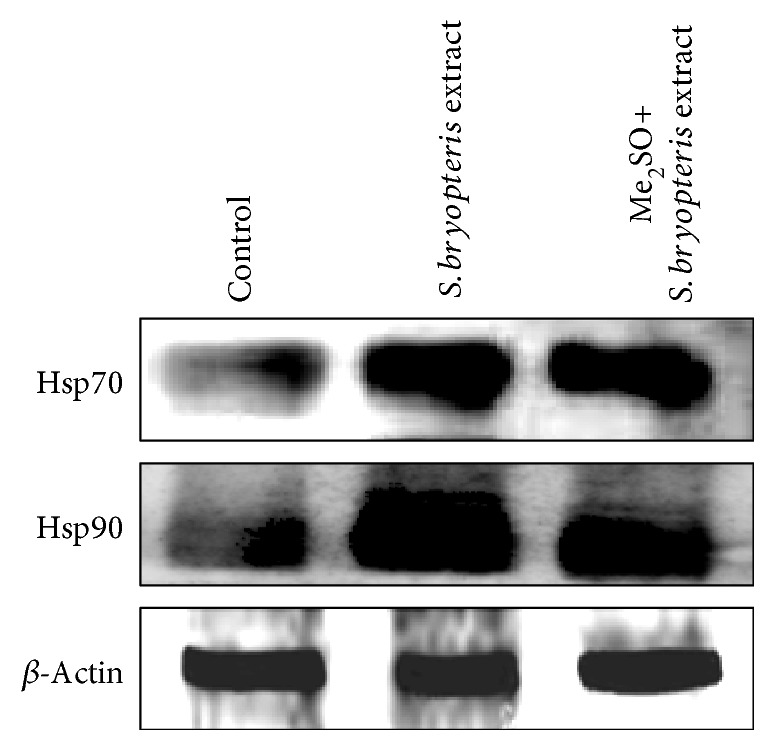
Western blot analysis for the expression of Hsp70 and Hsp90 in postthawed MSCs cryopreserved with Me_2_SO, *Selaginella bryopteris* water extract, and their combination.

To determine the potential involvement of the Hsps during postcryopreservation cell viability and functionality of MSCs, the expression analysis of Hsp70 and Hsp90 was carried out with Western immunoblotting. Western blot data demonstrated that the expression of Hsp70 and Hsp90 was upregulated in postthawed MSCs cryopreserved with *Selaginella bryopteris* water extract when compared with noncryopreserved control MSCs. Moreover, the combinatorial use of Me_2_SO and *Selaginella bryopteris* water extract in freezing medium further downregulated the expression of Hsp70 and Hsp90 proteins as compared to *Selaginella bryopteris* water extract alone (Figure 6()). The study indicated that the expression of heat shock proteins correlated positively with MSC viability. Additionally, the expressions of Hsp70 and Hsp90 proteins were the most significant in postthawed MSCs cryopreserved in freezing medium containing the combination of *Selaginella bryopteris* water extract and Me_2_SO. In the absence of a cryoprotectant, the MSCs showed poor expression of heat shock proteins. Thus, higher expression of Hsp70 and Hsp90 could probably lead to the higher cell viability and freezing resistance of MSCs after a freezing-thawing process. Thus, we concluded that the increased level of heat shock proteins in the presence of the water-soluble extract of *Selaginella bryopteris* could be used to predict reliably the freezing resistance of MSCs.

## 4. Discussion

MSCs offer an important cell source for regenerative medicines and specific tissue engineering. However, the successful therapeutic application of MSCs relies on the development of an appropriate cryopreservation protocol that ensures desired cell viability, metabolic activity, and plasticity potential of MSCs upon long-term storage. Although the conventional cryopreservation method using Me_2_SO is effective, cell viability and cell recovery of MSCs cryopreserved with Me_2_SO were always remained a critical issue due to Me_2_SO-induced cytotoxicity and neurodifferentiation in preserved MSCs [8, 9].

In the present study, we analyzed the effectiveness of the cryopreservation procedure using the water-soluble extract of *Selaginella bryopteris*. The maximum viability and cryopreservation potential were obtained when MSCs were cryopreserved in freezing medium containing both Me_2_SO and *Selaginella bryopteris* water extract. We subsequently evaluated the effect of *Selaginella bryopteris* water extract in the presence and absence of Me_2_SO on cell morphology, cell viability, cell attachment, and expression of heat shock proteins (Hsps) in postthawed MSCs.

Previously, we formulated a freezing medium containing trehalose, ectoine, and catalase for effective cryopreservation, and encouraging results were obtained with human umbilical cord blood (hUCB) mononuclear cells [30, 31]. In the present study, the work has been further extended to investigate the effect of *Selaginella bryopteris* water extract with and without Me_2_SO as an effective cryoprotective solution for the cryopreservation of hUCB-derived MSCs. Thus, the present study deals with a reduction in Me_2_SO concentration as low as possible in freezing medium as well as an addition of natural osmolytes to avoid the cytotoxic effect of Me_2_SO and achieve improved recovery of MSCs after long-term storage. The overproduction of reactive oxygen species (ROS) during hypothermia and freezing is another important factor that causes free radical-mediated oxidative damage to macromolecules leading to the loss of a cell viability during and after freezing. An addition of suitable antioxidant in freezing medium is another important factor that helps to overcome the oxidative damage to the cells for maximum recovery during cryopreservation [31]. The water-soluble extract of *Selaginella bryopteris* acts as a potential antioxidant when added to the freezing medium as reported earlier [17]. Thus, *Selaginella bryopteris* water extract was investigated to assess its effectiveness as a potential antioxidant in the present study which was found to exert a beneficial effect on the recovery of cryopreserved cells by efficiently scavenging freezing-induced elevated level of ROS in MSCs.

In the first phase of the study, the major aim was to determine the effect of *Selaginella bryopteris* water-soluble extract on cell viability of MSCs during cryopreservation. The cell viability of MSCs after cryopreservation was around 40% with *Selaginella bryopteris* water extract and 61% with Me_2_SO. The inclusion of *Selaginella bryopteris* water extract as cryoprotecting solution increases the viability and overall recovery of the hUCB MSCs. Thus, the cell viability data demonstrated the improved recovery of MSCs with *Selaginella bryopteris* water extract, suggesting a possibility of its use as an additive mixture during cryopreservation.

Next, we assessed the apoptotic and necrotic cell death of MSCs cryopreserved with *Selaginella bryopteris* water extract in the presence and absence of Me_2_SO, and the data demonstrated that *Selaginella bryopteris* water extract protected the cells against freezing-induced apoptosis. Recent studies have reported an elevation in ROS like superoxide anion and hydrogen peroxide during the cryopreservation procedure [31]. The elevated level of superoxide anion induces the release of cytochrome c from the intermembrane of mitochondria into the cytoplasm that eventually leads to apoptotic cell death. Therefore, ROS scavenging using strong antioxidants can be an effective strategy for improving cell recovery during cryopreservation. Interestingly, the use of *Selaginella bryopteris* water extract in combination with Me_2_SO led to a significant reduction in ROS generation. Moreover, the prevention of ROS generation in cryopreserved MSCs by *Selaginella bryopteris* water extract indicates its involvement in the maintenance of mitochondrial health. The overproduction of ROS is a common phenomenon due to the occurrence of cryopreservation-induced stress. Thus, the study confirmed that the supplementation of *Selaginella bryopteris* water-soluble extract has a significant positive effect on mitochondrial health in postthawed MSCs.

The cytoskeleton such as filamentous actin performs multitude of functions such as maintenance of cell shape, muscle contraction, cell signalling regulating cell dynamics, and motility. Moreover, involvement of different proteins can also alter the organization and distribution of the cytoskeleton. The cytoskeleton integrity plays a major role in cell viability, proliferation, and differentiation. Loss of function of the cell membrane interferes with transport systems such as pH regulatory systems of the cell. Disruption of organelle membranes affects transport systems such as mitochondrial transport systems essential for oxidative phosphorylation, the major energy generating pathway [24]. Therefore, cytoskeleton integrity was assessed by confocal microscopy in the present investigation. It was evident that the noncryopreserved MSCs have retained their cell morphology with the intact cell membrane. Immunofluorescence shows that the cell attachment was less in cryopreserved MSCs as compared to noncryopreserved MSCs irrespective of freezing solution. Moreover, it has also been demonstrated that the freezing medium containing the combination of *Selaginella bryopteris* water extract and Me_2_SO improved the cell viability and exhibited maximum retention of the cytoskeleton of cryopreserved and postthawed MSCs.

As a protective response to external stimuli, Hsps increase their expression, help to maintain the metabolic and structural integrity of the cells, and enable the cells to become more resistant to stress conditions [32–34]. It is also suggested that Hsps inhibit apoptotic cell death [32]. Hsp70 [33] and Hsp90 [34] in combination or alone play a major role in morphogenesis and dimorphism [35]. Protein denaturation has been reported in stress conditions and during modulation in temperature, which causes native misfolded aggregation of proteins that ultimately leads to the loss of biological functions as well as apoptosis [36]. The stress-related changes are responded by a set of proteins known as Hsps, which facilitate survival of the organism. The expressions of Hsps are found ubiquitously in the cell membrane, cytoplasm, and various other cell organelles such as mitochondria, endoplasmic reticulum, and nucleus. The pivotal role of Hsps involves the regulation of cell cycle progression, DNA replication, and transcriptional and posttranslational processes such as protein folding, stability, transportation, and degradation [37]. Moreover, they are also reported in the activation of many key signal transducers in animal cells. Hsps are highly conserved biomolecules which are constitutively expressed and upregulated in response to various stress stimuli. It is also suggested that Hsps play an important role in the maintenance of cellular homeostasis under the influence of stress conditions [38]. In this study, we assessed and compared the expressions of Hsp70 and Hsp90 in MSCs before and after cryopreservation and also explored the relationship among the expressions of Hsps in MSCs in the presence of the water-soluble extract of *Selaginella bryopteris* and associated protection of the cells against toxicity exerted by the use of a cryoprotective agent such as Me_2_SO as well as against the freezing-thawing injury. The results showed that the levels of Hsp70 and Hsp90 were significantly higher in MSCs cryopreserved in freezing medium containing *Selaginella bryopteris* water extract alone or in combination with Me_2_SO when compared with noncryopreserved MSCs. Thus, our data substantiate that *Selaginella bryopteris* aqueous extract improves stability and function of cryopreserved hUCB-derived MSCs.

## Figures and Tables

**Table 1:** Apoptosis study in postthawed MSCs and percentage change in MSC population in different phases of apoptotic cell death.

	Live cells	Early apoptotic cells	Late apoptotic cells	Necrotic cells
Control	80.59	14.16	3.66	1.59
*S. bryopteris* water extract	44.76	18.10	26.76	10.38
Me_2_SO	75.56	22.94	1.17	0.33
Me_2_SO + *S. bryopteris* water extract	75.40	12.99	9.96	1.65

## References

[1] Giordano A., Galderisi U., Marino I. R. (2007). From the laboratory bench to the patient’s bedside: an update on clinical trials with mesenchymal stem cells. *Journal of Cellular Physiology*.

[2] D’souza N., Burns J. S., Grisendi G. (2012). MSC and tumors: homing, differentiation, and secretion influence therapeutic potential. *Advances in Biochemical Engineering/Biotechnology*.

[3] Marquez-Curtis L. A., Janowska-Wieczorek A., McGann L. E., Elliott J. A. W. (2015). Mesenchymal stromal cells derived from various tissues: biological, clinical and cryopreservation aspects. *Cryobiology*.

[4] Sasaki M., Honmou O. (2017). Mesenchymal stem cells. *Cell Therapy against Cerebral Stroke*.

[5] Gramlich O. W., Burand A. J., Brown A. J., Deutsch R. J., Kuehn M. H., Ankrum J. A. (2016). Cryopreserved mesenchymal stromal cells maintain potency in a retinal ischemia/reperfusion injury model: toward an off-the-shelf therapy. *Scientific Reports*.

[6] Ginani F., Soares D. M., Barboza C. A. (2012). Effect of a cryopreservation protocol on the in vitro yield of adipose-derived stem cells. *Revista Brasileira de Cirurgia Plástica*.

[7] Luo K., Wu G., Wang Q., Sun Y., Liu H. (1994). Effect of dimethylsulfoxide and hydroxyethyl starch in the preservation of fractionated human marrow cells. *Cryobiology*.

[8] Bissoyi A., Pramanik K. (2013). Effects of non-toxic cryoprotective agents on the viability of cord blood derived MNCs. *Cryoletters*.

[9] Jain K. G., Mohanty S., Ray A. R., Malhotra R., Airan B. (2015). Culture and differentiation of mesenchymal stem cell into osteoblast on degradable biomedical composite scaffold: in vitro study. *The Indian Journal of Medical Research*.

[10] Hunt C. J. (2017). Cryopreservation: vitrification and controlled rate cooling. *Methods in Molecular Biology*.

[11] Yuan Z., Lourenco S. D. S., Sage E. K., Kolluri K. K., Lowdell M. W., Janes S. M. (2016). Cryopreservation of human mesenchymal stromal cells expressing TRAIL for human anti-cancer therapy. *Cytotherapy*.

[12] Varma V. P., Devi L., Venna N. K., Murthy C. L. N., Idris M. M., Goel S. (2015). Ocular fluid as a replacement for serum in cell cryopreservation media. *PloS One*.

[13] Dogan A., Yalvac M. E., Yılmaz A., Rizvanov A. A., Sahin F. (2013). Effect of f68 on cryopreservation of mesenchymal stem cells derived from human tooth germ. *Applied Biochemistry and Biotechnology*.

[14] Yalvac M. E., Ramazanoglu M., Tekguc M. (2010). Human tooth germ stem cells preserve neuro-protective effects after long-term cryo-preservation. *Current Neurovascular Research*.

[15] Mishra P. K., Raghuram G. V., Bhargava A. (2011). In vitro and in vivo evaluation of the anticarcinogenic and cancer chemopreventive potential of a flavonoid-rich fraction from a traditional Indian herb Selaginella bryopteris. *The British Journal of Nutrition*.

[16] Gechev T. S., Hille J., Woerdenbag H. J. (2014). Natural products from resurrection plants: potential for medical applications. *Biotechnology Advances*.

[17] Sah N. K., Singh S. N. P., Sahdev S. (2005). Indian herb “*Sanjeevani*” (*Selaginella bryopteris*) can promote growth and protect against heat shock and apoptotic activities of ultra violet and oxidative stress. *Journal of Biosciences*.

[18] Chandran G., Muralidhara (2014). Insights on the neuromodulatory propensity of Selaginella (Sanjeevani) and its potential pharmacological applications. *CNS & Neurological Disorders - Drug Targets*.

[19] Kunert O., Swamy R. C., Kaiser M. (2008). Antiplasmodial and leishmanicidal activity of biflavonoids from Indian Selaginella bryopteris. *Phytochemistry Letters*.

[20] Haack-Sørensen M., Kastrup J. (2011). Cryopreservation and revival of mesenchymal stromal cells. *Methods in Molecular Biology*.

[21] Crook J. M., Tomaskovic-Crook E. (2017). Culturing and cryobanking human neural stem cells. *Methods in Molecular Biology*.

[22] Pavón A., Beloqui I., Salcedo J. M., Martin A. G. (2017). Cryobanking mesenchymal stem cells. *Methods in Molecular Biology*.

[23] Lyons T. A., Amouretti X. F., Held P. G., Naleway J. J. (2008). Development of a live-cell based reactive oxygen species (ROS) assay for use in high-content screening of drug candidates using the BioTek synergy mx microplate reader.

[24] Dalcin L., Silva R. C., Paulini F., Silva B. D. M., Neves J. P., Lucci C. M. (2013). Cytoskeleton structure, pattern of mitochondrial activity and ultrastructure of frozen or vitrified sheep embryos. *Cryobiology*.

[25] Roato I., Alotto D., Belisario D. C. (2016). Adipose derived-mesenchymal stem cells viability and differentiating features for orthopaedic reparative applications: banking of adipose tissue. *Stem Cells International*.

[26] Bellagamba B. C., Abreu B. R. R. d., Grivicich I. (2016). Human mesenchymal stem cells are resistant to cytotoxic and genotoxic effects of cisplatin in vitro. *Genetics and Molecular Biology*.

[27] Death C., Cells S. (2014). Role of the apoptosis pathway in cryopreservation-induced from umbilical cord blood. *Biopreservation and Biobanking*.

[28] Singh A. K., Bissoyi A., Kashyap M. P., Patra P. K., Rizvi S. I. (2017). Autophagy activation alleviates amyloid-*β*-induced oxidative stress, apoptosis and neurotoxicity in human neuroblastoma SH-SY5Y cells. *Neurotoxicity Research*.

[29] Behera S. S., Das U., Kumar A., Bissoyi A., Singh A. K. (2017). Chitosan/TiO_2_ composite membrane improves proliferation and survival of L929 fibroblast cells: application in wound dressing and skin regeneration. *International Journal of Biological Macromolecules*.

[30] Bissoyi A., Bit A., Singh B. K., Singh A. K., Patra P. K. (2016). Enhanced cryopreservation of MSCs in microfluidic bioreactor by regulated shear flow. *Scientific Reports*.

[31] Bissoyi A., Kumar A., Rizvanov A. A. (2016). Recent advances and future direction in lyophilisation and desiccation of mesenchymal stem cells. *Stem Cells International*.

[32] Bradley E., Bieberich E., Mivechi N. F., Tangpisuthipongsa D., Wang G. (2012). Regulation of embryonic stem cell pluripotency by heat shock protein 90. *Stem Cells*.

[33] Fan G. C. (2012). Role of heat shock proteins in stem cell behavior. *Progress in Molecular Biology and Translational Science*.

[34] Chen Y. B., Lan Y. W., Hung T. H. (2015). Mesenchymal stem cell-based HSP70 promoter-driven VEGFA induction by resveratrol promotes angiogenesis in a mouse model. *Cell Stress & Chaperones*.

[35] Gao F., Hu X., Xie X. (2010). Heat shock protein 90 protects rat mesenchymal stem cells against hypoxia and serum deprivation-induced apoptosis via the PI3K/Akt and ERK1/2 pathways. *Journal of Zhejiang University Science. B*.

[36] Munje C., Shervington L., Khan Z., Shervington A. (2014). Could upregulated Hsp70 protein compensate for the Hsp90-silence-induced cell death in glioma cells?. *International Journal of Brain Science*.

[37] Park J. A., Kim Y. E., Seok H. J., Park W. Y., Kwon H. J., Lee Y. H. (2011). Differentiation and upregulation of heat shock protein 70 induced by a subset of histone deacetylase inhibitors in mouse and human embryonic stem cells. *BMB Reports*.

[38] Chang W., Song B. W., Lim S. (2009). Mesenchymal stem cells pretreated with delivered Hph-1-Hsp70 protein are protected from hypoxia-mediated cell death and rescue heart functions from myocardial injury. *Stem Cells*.

